# Effect of Vitamin D3 Supplementation on Severe COVID-19: A Meta-Analysis of Randomized Clinical Trials

**DOI:** 10.3390/nu16101402

**Published:** 2024-05-07

**Authors:** Marharyta Sobczak, Rafał Pawliczak

**Affiliations:** Department of Immunopathology, Division of Biomedical Science, Faculty of Medicine, Medical University of Lodz, 90-752 Lodz, Poland

**Keywords:** vitamin D3, COVID-19, coronavirus infection, supplementation, meta-analysis

## Abstract

Since the beginning of the COVID-19 pandemic, vitamin D has attracted interest due to its immunomodulatory properties. Numerous studies show a correlation between vitamin D levels and COVID-19 cases and mortality. Therefore, we conducted a meta-analysis in order to assess the relationship between vitamin D3 supplementation and COVID-19 severity. We included 13 randomized clinical trials that contained the analyzed endpoints: length of COVID-19 hospitalization, number of intensive care unit (ICU) admissions, length of stay in the ICU, number of cases requiring any supplemental oxygenation, duration of any supplemental oxygenation, number of overall mortality and number of deaths associated with COVID-19. The relative risk with 95% confidence interval (CI) and the mean difference with 95% CI were calculated to compare the effect. A random effects model was used to calculate effect sizes. Our meta-analysis showed a positive effect of vitamin D3 supplementation on ICU admission (RR = 0.73; 95% CI [0.57; 0.95], *p* = 0.02, *I*^2^ = 19.6%) and mortality associated with COVID-19 among patients (RR = 0.56; 95% CI [0.34; 0.91]; *p* = 0.02; *I*^2^ = 0%). Vitamin D3 supplementation may potentially reduce the risk of ICU admission and death associated with COVID-19.

## 1. Introduction

At the end of 2019, the coronavirus infection began in Wuhan, China, and spread rapidly throughout the world [1]. This pandemic has had a serious impact on healthcare systems worldwide [2]. COVID-19 (coronavirus disease 2019) is caused by a highly contagious RNA virus—SARS-CoV-2 (severe acute respiratory syndrome coronavirus 2). According to WHO [3], as of 25 February 2024, 7,035,337 COVID-19 deaths have been reported. Nonsynonymous mutations that arise in the S protein lead to increased viral transmission, replication and binding affinity of ACE2, as well as variability among different SARS-CoV-2 variants [4].

Besides the flu-like and gastrointestinal symptoms, COVID-19 patients develop shortness of breath and tachypnoea with worsening of the respiratory disease, and damage of lung tissue up to the development of acute respiratory distress syndrome (ARDS) [1,5]. ARDS may potentially lead to the development of septic shock. This, in turn, is associated with hospitalization in an intensive care unit (ICU) as well as mortality in COVID-19 patients over 60 years of age [1]. Moreover, in the case of severe coronavirus disease, comorbidities for example cardiovascular disease, hypertension, chronic obstructive pulmonary disease and diabetes are common [5]. 

Vitamin D has been extensively researched for positive clinical impact, and it has been proven that its supplementation is beneficial in a number of diseases, such as type 2 diabetes mellitus, obesity, rheumatoid arthritis and chronic obstructive pulmonary disease [6]. Of note, vitamin D acts as an immunomodulator and antimicrobial agent [7]. Vitamin D is expected to stimulate the release of cathelicidin antimicrobial polypeptide as well as defensins, which counteract viral infections. Moreover, it suppresses the angiopoietin–Tie2 pathway, reducing cytokine secretion and, thus, hampering the cytokine storm [8]. SARS-CoV-2 may attack through its effect on cytokine components. Moreover, high levels of pro-inflammatory cytokines induce severe COVID-19 complications such as ARDS [9]. Vitamin D is a negative regulator of the renin–angiotensin–aldosterone system that contains the ACE2 receptor, which is expressed in the kidneys, heart, intestinal cells and lungs and is correlated with enhanced SARS-CoV-2 infection. Moreover, vitamin D affects oxidative stress, and thus, the fusion of the virus protein with the ACE2 receptor because reactive oxygen species favor the interaction of SARS-CoV-2 S protein with this receptor via the formation of disulfide bonds [10].

It was shown that a considerable percentage of patients who experienced severe COVID-19 symptoms were vitamin D deficient [8]. Interestingly, low levels of this vitamin were associated with the development of ARDS, lung damage and increased risk of severe COVID-19-related complications such as cardiovascular events [11].

In 2020, different recommendations regarding vitamin D supplementation in the face of the pandemic emerged. Since there have been no clinical studies yet on the role of such supplementation in relation to COVID-19, the recommendations focused on maintaining bone and muscle health [12,13]. COVID-19 treatment guidelines from the National Institutes of Health [14], which were updated on December 2023, described that there were no sufficient data to advise or prohibit supplementation for COVID-19 prevention and treatment. Considering the abovementioned factors, we decided to conduct a meta-analysis of control-compared randomized clinical trials in order to assess the correlation between vitamin D3 supplementation and COVID-19 severity.

## 2. Materials and Methods

### 2.1. Search Strategy

This meta-analysis was conducted according to the Preferred Reporting Items for Systematic Reviews and Meta-Analyses (PRISMA) guidelines [15]. PubMed, Web of Science, Embase and the Cochrane Central Register of Controlled Trials databases were searched to find literature published as of 15 February 2024. The following keywords were used: “Vitamin D”, “Vitamin D3”, “25-hydroxyvitamin D”, “Cholecalciferol”, “25(OH)D”, “supplement”, “supplementation”, “COVID-19”, “coronavirus infection”, “SARS-CoV-2”, “severe”, “severity”.

### 2.2. Study Selection and Data Extraction

In the study, we included only articles describing control-compared RCTs that took up the subject of vitamin D3 supplementation in patients suffering from severe COVID-19. Moreover, studies that either were not written in English and/or did not investigate endpoints such as length of COVID-19 hospitalization, number of ICU admissions, length of stay in the ICU, number of cases requiring any supplemental oxygenation, duration of any supplemental oxygenation, number of overall mortality and number of deaths associated with COVID-19 were excluded from our study.

Continuous data were converted into mean (SD):

- If the data were presented as median (Q1–Q3), the values were converted according to the method presented by Luo et al. [16] and Wan et al. [17] using the available calculator without checking the skewness.
- If the data were presented as median (IQR), Q1 and Q3 were calculated as median ± (IQR/2) and then the values were converted as mentioned above.

If the number of cases was given as a percentage, it was converted to whole numbers according to the rounding rules.

### 2.3. Quality Assessment

The study quality and risk of bias analyses were conducted in compliance with the Cochrane Collaboration’s tool for assessing risk of bias in randomized trials [18]. The following criteria were used in order to assess the quality and bias of included studies: random sequence generation, allocation concealment, blinding of participants and personnel, blinding of outcome assessment, incomplete outcome data, selective reporting and other biases. All of the criteria were assessed on the three levels—low, high, or unclear risk of bias.

### 2.4. Statistical Analysis

Statistical analyses of data were conducted in the R environment (version 4.2.2). In order to compare the effect of vitamin D3 supplementation in the treatment group and the control, the relative risk (RR), with a 95% confidence interval (CI), was calculated for dichotomous outcomes, while the mean difference, with 95% CI, was calculated for continuous outcomes. The effect size of analyzed variables was calculated using the random effects model. The heterogeneity was assessed using *I*^2^ statistics. The *I*^2^ values were interpreted as follows: *I*^2^ < 40% may not be relevant; 30% < *I*^2^ < 60%—moderate heterogeneity; 50% < *I*^2^ < 90%—substantial heterogeneity; *I*^2^ > 75%—considerable heterogeneity [19]. The publication bias was evaluated using funnel plots, Peters’ regression test (for dichotomous outcomes) and Egger’s regression test (for continuous outcomes). The level of statistical significance of the meta-analysis was set at the *p* < 0.05.

## 3. Results

### 3.1. Search Results

**Figure 1:**
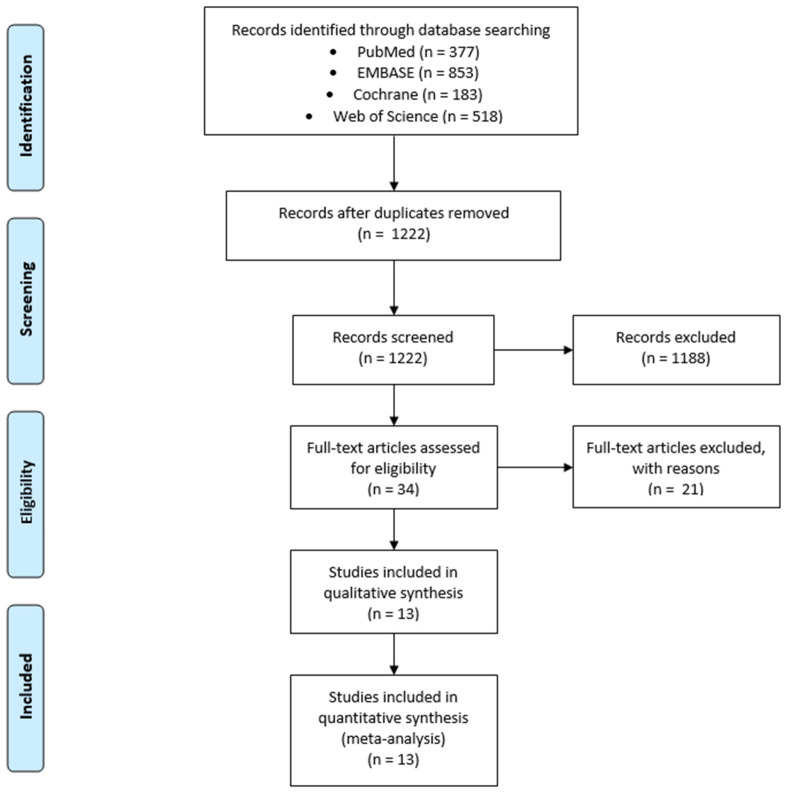
Selection of studies for meta-analysis.

The literature search resulted in 1222 articles after the removal of duplicates (Figure 1). During the first screening, we excluded 1188 articles, including meta-analyses, systematic reviews, literature reviews and editorials, as well as in vitro studies, studies on animals, case reports, observational studies and quasi-experimental studies. Moreover, we included articles written only in English. After full-text screening, 13 articles qualified for analysis.

All included studies are randomized controlled trials with a control group containing vitamin D3 supplementation. Among these studies, five studies had a placebo in the control group [20,21,22,23,24], four studies had no supplementation in the control group [25,26,27,28] and four studies had a standard dose of vitamin D3 [29,30,31,32]. Two studies included patients admitted to the intensive care unit [21,32]. One study included pediatric patients [31] and one study enrolled older adults [26]. The studies were carried out in different countries, including Spain [28,29], Brazil [20], Russia [21,30], Switzerland [27], Mexico [31], Egypt [25], France [26], Argentina [22], Belgium [23], Iran [24] and Croatia [32]. Table 1 shows the characteristics of the included studies.

### 3.2. Quality Assessment

Risk of bias analysis was conducted for 13 included RCTs. According to the analysis, in 4 of the assessed studies, there was a high risk of bias, while in the remaining 9 studies, the risk was relatively low. Figure S1 shows the summary of the risk of bias assessment.

### 3.3. Effect of Vitamin D3 Supplementation on Length of COVID-19 Hospitalization

**Figure 2:**
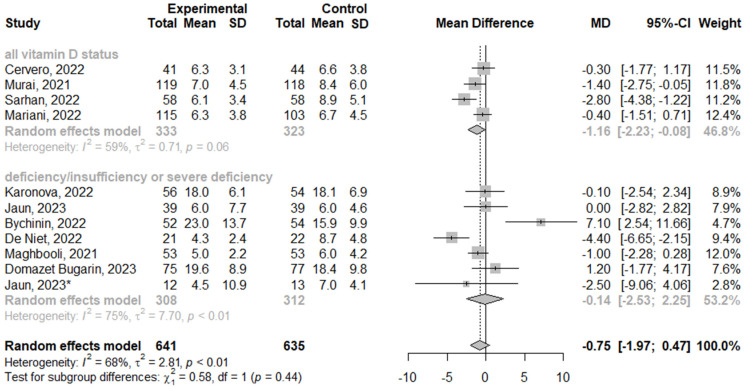
The efficacy of vitamin D3 supplementation on the length of COVID-19 hospitalization. * Severe deficiency status of vitamin D [20,21,22,23,24,25,27,28,30,32].

We analyzed the effect of vitamin D3 supplementation on the length of COVID-19 hospitalization using the subgroup analysis of clinical trials. Overall, the analysis showed no difference between vitamin D3 supplementation and control groups (MD = −0.78; 95% CI [−1.97; 0.47]; *p* = 0.23; *I*^2^ = 68.2%), as shown in Figure 2.

### 3.4. Effect of Vitamin D3 Supplementation on Number of ICU Admissions and Length of Stay in the ICU

**Figure 3:**
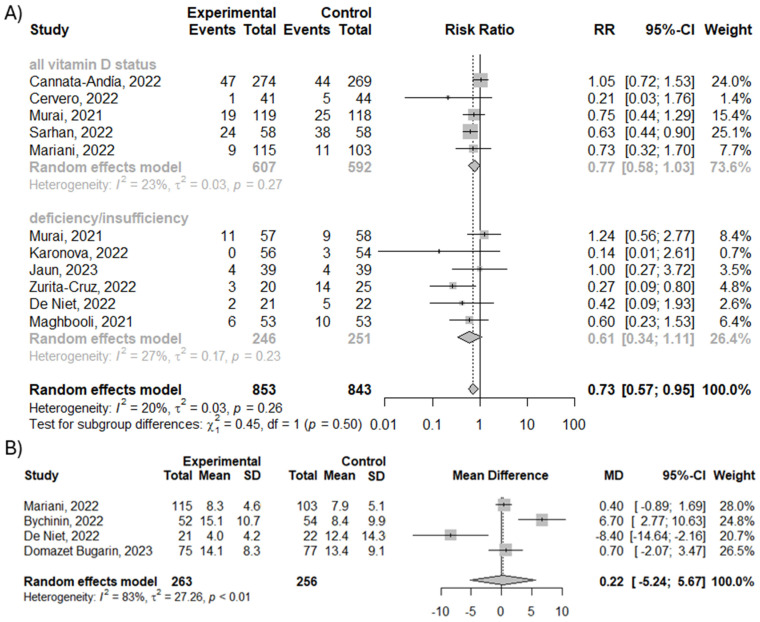
(**A**) The efficacy of vitamin D3 supplementation on the number of ICU admissions. (**B**) Mean difference in length of stay in the ICU [20,21,22,23,24,25,27,28,29,30,31,32].

We analyzed the effect of vitamin D3 supplementation on the number of ICU admissions and the length of stay in the ICU. Overall, subgroup analysis showed that supplementation with vitamin D3 reduces the risk of ICU admission by 27% (RR = 0.73; 95% CI [0.57; 0.95], *p* = 0.02, *I*^2^ = 19.6%), with no differences between analyzed groups (*p* = 0.50), as shown in Figure 3A. However, the analysis showed no difference between vitamin D3 supplementation and control groups in case of length of stay in the ICU (MD = 0.22; 95% CI [−5.24; 5.67]; *p* = 0.94; *I*^2^ = 82.7%), as shown in Figure 3B.

### 3.5. Effect of Vitamin D3 Supplementation on Number of Cases Requiring Supplemental Oxygenation and Duration of Supplemental Oxygenation

**Figure 4:**
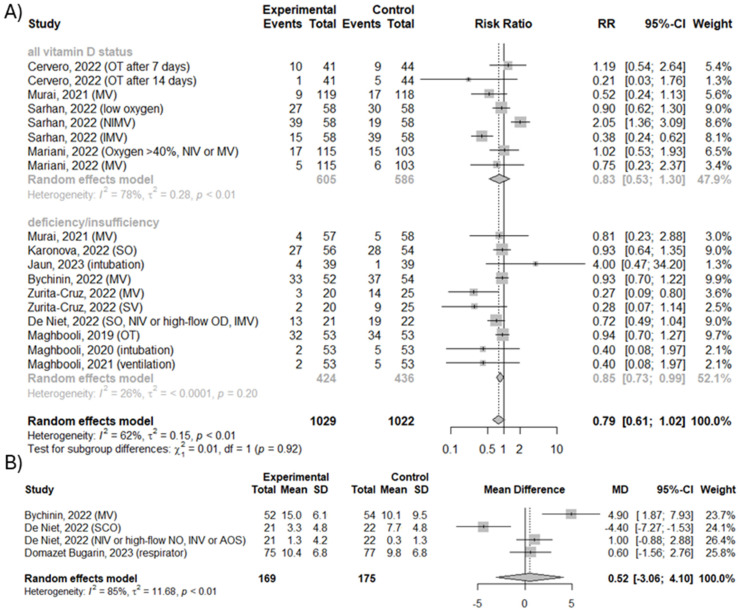
(**A**) The efficacy of vitamin D3 supplementation on number of cases requiring any supplemental oxygenation. (**B**) Mean difference in duration of any supplemental oxygenation. OT—oxygen therapies; MV—mechanical ventilation; NIMV or NIV—non-invasive mechanical ventilation or non-invasive ventilation; IMV—invasive mechanical ventilation; OD—oxygen devices; SO—supplemental oxygenation; SV—progression to a superior ventilation modality; SCO—supplemental conventional oxygen [20,21,22,23,24,25,27,28,30,31,32].

We also checked whether vitamin D3 supplementation influenced the number of cases requiring any supplemental oxygenation and the duration of any supplemental oxygenation. However, our subgroup analysis showed no significant effect of vitamin D3 supplementation on the number of cases requiring any supplemental oxygenation (RR = 0.79; 95% CI [0.61; 1.02], *p* = 0.07, *I*^2^ = 62.2%) (Figure 4A), as well as no differences between analyzed groups in case of duration of any supplemental oxygenation (MD = −0.52; 95% CI [−3.06; 4.10]; *p* = 0.78; *I*^2^ = 84.7%), as shown in Figure 4B.

### 3.6. Effect of Vitamin D3 Supplementation on Number of Overall Mortality and Number of Deaths Associated with COVID-19

**Figure 5:**
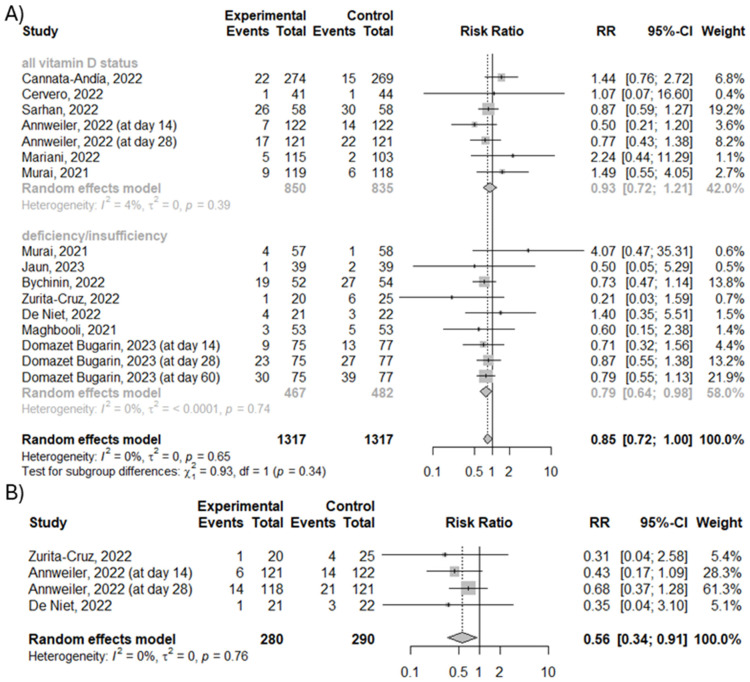
(**A**) The efficacy of vitamin D3 supplementation on the number of overall mortality. (**B**) The efficacy of vitamin D3 supplementation on the number of deaths associated with COVID-19 [20,21,22,23,24,25,26,27,28,29,31,32].

Vitamin D3 supplementation had no significant effect on the overall mortality of patients with COVID-19 (RR = 0.85; 95% CI [0.72; 1]; *p* = 0.051; *I*^2^ = 0%), as shown in Figure 5A. However, our meta-analysis showed that vitamin D3 supplementation may decrease the risk of death associated with COVID-19 in comparison to the control group by 44% (RR = 0.56; 95% CI [0.34; 0.91]; *p* = 0.02; *I*^2^ = 0%) (Figure 5B).

### 3.7. Publication Bias

The results of publication bias analysis for all the investigated outcomes, including length of COVID-19 hospitalization, number of ICU admissions, length of stay in the ICU, number of cases requiring any supplemental oxygenation, duration of any supplemental oxygenation, number of overall mortality and number of deaths related to COVID-19, are shown in the funnel plots (Figure S2). Moreover, in order to assess publication bias, Peters’ regression test and Egger’s regression test were performed. The results showed that there was no evidence of publication bias in the association between vitamin D3 supplementation and length of COVID-19 hospitalization (*p* = 0.47), length of stay in the ICU (*p* = 0.95), number of cases requiring any supplemental oxygenation (*p* = 0.24), duration of any supplemental oxygenation (*p* = 0.94), number of overall mortality (*p* = 0.19) and number of deaths associated with COVID-19 (*p* = 0.32). However, publication bias was present between vitamin D3 supplementation and number of ICU admissions (*p* = 0.02).

## 4. Discussion

Our meta-analysis of data collected from 13 randomized clinical trials showed a positive effect of vitamin D3 supplementation on ICU admission and mortality associated with COVID-19 among patients. However, vitamin D3 supplementation did not significantly influence the length of hospitalization due to COVID-19, length of stay in the ICU, requirement of any type of supplemental oxygenation and duration of any type of supplemental oxygenation, as well as overall mortality.

The currently available literature shows conflicting results on the topic of efficiency of vitamin D3 supplementation in terms of mortality and severe course of COVID-19. Results presented in multiple studies are in line with our data, although some authors report a lack of evidence in advocating for the beneficial effects of the supplementation. Similar results were observed in other meta-analyses, as a study based on 24 observational studies demonstrated that low levels of serum calciferol were correlated with mortality and pulmonary complications in the course of COVID-19. Moreover, vitamin D-deficient patients were more likely to develop cardiovascular COVID-19-related complications and had elevated levels of inflammation markers [33]. Another study pointed out a significant association between COVID-19 mortality, hospitalization rate and length of hospitalization and 25(OH)D deficiency [34]. Bassatne et al. [35] suggested that calciferol may have a beneficial impact in terms of mortality prevention, as vitamin D-deficient subjects were characterized by higher mortality rates, although the supplementation affected neither the length of hospitalization nor COVID-19 severity. A systematic review and meta-analysis conducted by Sîrbu et al. [36] indicated that calciferol supplementation did not affect the mortality rate, although it decreased ICU rate and hospitalization length among COVID-19 patients. Other meta-analyses demonstrated that supplementation with vitamin D was correlated with a decreased risk of COVID-19 infection, based on seven RCTs, and ICU admission, based on three analytical studies [37]. A retrospective, non-randomized cohort study showed that patients supplemented with vitamin D presented lower mortality as well as lower pneumonia severity scores compared with patients enrolled in the control group [38]. Interestingly, a combination of vitamin D, magnesium and vitamin B12 decreased the number of elderly COVID-19 patients who required supported ventilation and/or ICU admission [39]. An observational study conducted among United Arab Emirates citizens showed that lower levels of serum calcitriol were associated with higher mortality rates and SARS-CoV-2 infection severity [40].

On the other hand, a number of studies describe no interdependence between 25(OH)D levels and COVID-19-related outcomes. In the study by Rawat et al. [41], no significant impact of vitamin D in COVID-19 patients was observed. Vitamin D reduced neither mortality, ICU admission rate, nor the need for breathing support. Similarly, another study showed no correlation between vitamin D supplementation and COVID-19 mortality, ICU admission and the need for mechanical ventilation. Although some authors claimed that there was a trend toward reduced COVID-19 mortality following vitamin D administration [42], a large randomized controlled trial carried out on 6200 people in the United Kingdom demonstrated that vitamin D supplementation, either at a dose of 800 IU/day or at a dose of 3200 IU/day, had no effect on the prevention of acute respiratory tract infection as well as COVID-19 [43]. 

In terms of the efficacy of vitamin D supplementation against COVID-19, aside from the dosage, the repetitiveness of supplementation seems to be a crucial factor in reducing COVID-19-related mortality. In a randomized clinical study carried out in Tunisia, a high, single dose of vitamin D (200,000 IU) did not alter the period of hospitalization in comparison to the placebo group who received physiological saline [44]. However, an observational study conducted in the United Kingdom showed that different regimens of vitamin D3 supplementation, administered during the period of hospitalization, were successful in decreasing mortality among study participants who at the point of hospitalization were vitamin D deficient [45]. Similarly, in a double-blind, randomized trial, Villasis-Keever et al. [46] described the protective effect of vitamin D supplementation irrespective of basal vitamin D serum levels. A vitamin D dose of 4000 IU, administered for 30 days, was proven to decrease the risk of SARS-CoV-2 infection in medical professionals in comparison to the placebo (cornstarch tablet). Moreover, in a study by Entrenas Castillo et al. [47], a high dose of calcifediol decreased the requirement for ICU among COVID-19-hospitalized patients. A clinical case series of four patients with COVID-19 showed that a high dose of ergocalciferol (50,000 IU per day for 5 days) was better in relation to shorter stays in hospital, lower oxygen requirements and lowering the status of inflammatory markers in comparison to patients administered the standard dose of cholecalciferol (1000 IU per day) [48]. However, vitamin D2 supplementation is less effective than vitamin D3 supplementation at increasing the total concentration of 25(OH)D in serum, as shown by a study in which a bolus dose of both vitamins was used [49].

Our meta-analysis has some limitations. Various factors may influence the effectiveness of vitamin D supplementation, for example, sunlight exposure in the region. Moreover, comorbidities may affect vitamin D levels. At the same time, comorbidities, such as age over 65 years old, diabetes mellitus, obesity and hypertension, are important risk factors that may cause severe COVID-19 infection. A meta-analysis showed that obese patients were at a higher risk of contracting COVID-19 infection [50]. In our meta-analysis, a study involving people above 65 years of age who were supplemented with different single doses of cholecalciferol [26] detected the highest risk of death associated with COVID-19, while a study of participants who were less than 18 years old, among whom none had a normal serum levels of 25(OH)D [31], showed the lowest risk of COVID-19 mortality. Moreover, aging also influences vitamin D production because it decreases as a result of age-related deterioration of kidney function [51]. Moreover, not all included studies had placebo or no supplementation as a control group. There are also differences in the characteristics of participants in the included studies, such as severity of COVID-19 infection, vitamin D3 status and age. All these factors may lead to high heterogeneity in our results; therefore, we used subgroup analysis based on vitamin D3 status when the number of studies was sufficient. Of note, studies reported results using various summary statistics, thus their conversion into a common measure may have caused discrepancies. The data were transformed without checking for skewness to avoid excluding studies. Additionally, data presented as (range) or median [95% CI] were not included in our analysis due to the impossibility of transforming such data. Furthermore, the total number of people in all the studies combined is rather small compared to the very large number of people infected with COVID-19. Moreover, we detected publication bias in the context of the number of ICU admissions according to Peters’ regression test. As can be seen from the funnel plot (Figure S2B), there is a lack of small studies in which the RR is above 1, i.e., those studies that indicate a negative effect of supplementation.

## 5. Conclusions

The present meta-analysis suggests that vitamin D3 supplementation may reduce the risk associated with COVID-19 mortality and ICU admission among COVID-19 patients. It is undeniable that vitamin D3 supplementation has a beneficial effect on the course of COVID-19 infection. Nevertheless, larger clinical trials are required to fully confirm the effect of vitamin D3 in the context of COVID-19.

## Figures and Tables

**Table 1:** Basic characteristics of included studies.

Studies	Study Design	Participants	Mean Age (Mean, SD)	Sex (Girl/Female)	Baseline Concentration of Serum Vitamin D (ng/mL or nmol/L), Mean (SD)	Vitamin D3 Treatment	Final Results of the Quality Assessment
Cannata-Andía et al., 2022 [29]	a randomized, open-label, multicenter, international, clinical, independent trial	≥18 years old with moderate-–severe COVID-19 disease	I: 59.4 (15.7) C: 56.3 (16.4)	I: 33.9% C: 36.1%	I: 16.9 (7.6) ng/mL C: 16.6 (7.8) ng/mL	I: a single oral bolus of 100,000 IU of cholecalciferol C: nothing	low
Cervero et al., 2022 [28]	a multicenter, single-blinded, prospective, randomized pilot clinical trial	≥18 years old diagnosed with COVID-19 pneumonia	I: 66.6 (13.1) C: 59.7 (21.5)	I: 27% C: 32%	I: 15.3 (6.3) ng/mL C: 14.3 (6.2) ng/mL	I: 10,000 IU of cholecalciferol once daily for 14 days C: 2000 IU of cholecalciferol once daily for 14 days	high
Murai et al., 2021 [20]	a multicenter, double-blind, parallel-group, randomized, placebo-controlled trial	≥18 years old diagnosed with COVID-19	I: 56.5 (13.8) C: 56.0 (15.0)	I: 41.2% C: 46.6%	I: 21.2 (10.1) ng/mL C: 20.6 (8.1) ng/mL	I: a single oral dose of 200,000 IU of vitamin D3 C: placebo	low
Karonova et al., 2022 [30]	a randomized single-center, open-label study	18–75 years old, confirmed diagnosis of COVID-19 with vitamin D insufficiency and deficiency	I: 57.6 (11.4) C: 62.9 (11.4)	I: 47.7% C: 50%	I: 16.4 (8.2) ng/mL C: 13.7 (5.9) ng/mL	I: a bolus of 50,000 IU of cholecalciferol on the 1st and the 8th days of hospitalization C: no supplementation	high
Jaun et al., 2023 [27]	a multicenter, randomized, placebo-controlled double-blind trial	>18 years old, ongoing COVID-19 infection with serum 25(OH)D <20 ng/mL	I: 60.5 (13.8) C: 61.4 (15.3)	I: 14% C: 7%	I: 31.5 (11) nmol/L C: 28.5 (10.1) nmol/L	I: a single dose of 140,000 IU (3500 μg) of vitamin D3 C: 800 of vitamin D3	low
Bychinin et al., 2022 [21]	a prospective, single-center, randomized, placebo-controlled pilot trial	≥18 years old with confirmed COVID-19 and admitted to the intensive care unit with serum 25(OH)D below 30 ng/mL	I: 64.1 (10.7) C: 66.3 (20.6)	I: 58% C: 43%	I: 12.2 (11.7) ng/mL C: 11.6 (4.9) ng/mL	I: 60,000 IU of cholecalciferol on days 1, 8, 16, 24 and 32; daily maintenance doses of 5000 IU of cholecalciferol on other days C: placebo	low
Zurita-Cruz et al., 2022 [31]	an open-label, randomized, controlled, single-blind clinical trial	younger than 18 years old with confirmed SARS-CoV-2 infection	I: 9.8 (8.1) C: 11.9 (5.9)	I: 55% C: 64%	I: 14.3 (6.1) ng/mL C: 11.04 (3.5) ng/mL	I: 1000 IU/day of vitamin D for children < 1 year and 2000 IU/day of vitamin D for children 1–17 years during hospitalization for a minimum of 7 days and a maximum of 14 days C: without supplementation	low
Sarhan et al., 2022 [25]	a prospective randomized controlled study	≥18 years old with a verified COVID-19 hyperinflammation status. I: moderate and severe SARS-CoV-2-infected patients C: SARS-CoV-2-infected patients	I: 66.1 (11.2) C: 65.7 (12.6)	I: 34.5% C: 20.7%	NA	I: a single high-dose vitamin D cholecalciferol (200,000 IU) C: a standard dose of alfacalcidol (1 µg/day) administered orally (as a standard of care during COVID-19 management)	high
Annweiler et al., 2022 [26]	An investigator-initiated, multicenter, open-label, parallel-group, intent-to-treat, randomized controlled superiority clinical trial	65 years or older, SARS-CoV-2 infection diagnosed	I: 86.6 (8.2) C: 88.3 (7.5)	I: 52% C: 65%	I: 54.4 (43.5) nmol/L C: 45.5 (30.8) nmol/L	I: a single oral dose of 400,000 IU of cholecalciferol C: a single oral dose of 50,000 IU of cholecalciferol	high
Mariani et al., 2022 [22]	a multicentre, randomized, double-blind, sequential, placebo-controlled trial,	≥18 years old with SARS-CoV-2 confirmed infection	I: 59.8 (10.7) C: 58.3 (10.6)	I: 44.3% C: 50.5%	I: 34.8 (12.8) ng/mL C: 29.7 (10.3) ng/mL	I: a single oral dose of 500,000 IU of vitamin D3 C: placebo	low
De Niet et al., 2022 [23]	an interventional, randomized, parallel, two-treatment, two-arm, double-blind and placebo-controlled pilot study	≥18 years old with vitamin D deficiency (≤20 ng/mL) and confirmed SARS-CoV-2 infection	I: 63.2 (14.5) C: 68.7 (11)	I: 38% C: 54%	I: 17.9 (10.2) ng/mL C: 16.9 (9.5) ng/mL	I: a daily dose of 25,000 IU of vitamin D (cholecalciferol) over four consecutive days, then, 25,000 IU per week up to six weeks C: placebo	low
Maghbooli et al., 2021 [24]	a randomized, double-blinded, placebo-controlled trial.	≥18 years old with vitamin D deficiency/insufficiency (<30 ng/mL) and diagnosed with COVID-19	I: 50 (15) C: 49 (13)	I: 41% C: 38%	I: 19 (8) ng/mL C: 18 (8) ng/mL	I: 25 μg of calcifediol once daily C: placebo	low
Domazet Bugarin et al., 2023 [32]	a single-center, open-label, randomized clinical trial	≥18 years old with low levels of vitamin D (<50 nmol/L) and confirmed COVID-19 disease who were admitted to the ICU	I: 65 (59–71) * C: 65.5 (39–82) *	I: 30.7% C: 25%	I: 26.8 (14.4) nmol/L C: 26.8 (16.1) nmol/L	I: 10,000 IU of cholecalciferol daily during ICU stay or for at least 14 days; if vitamin D levels were >150 nmol/L or calcium levels were >2.6 mmol/L, further supplementation was stopped C: without supplementation	low

NA—not applicable; I—intervention group; C—control group; * mean (min–max).

## Data Availability

The original contributions presented in the study are included in the article/Supplementary Material, further inquiries can be directed to the corresponding author.

## Supplementary Materials

The following supporting information can be downloaded at: https://www.mdpi.com/article/10.3390/nu16101402/s1, Figure S1: Risk of bias in included studies, Figure S2: Funnel plots of the association between vitamin D3 supplementation and COVID-19.
