# Supplementary material for: Effect of Vitamin D3 Supplementation on Severe COVID-19: A Meta-Analysis of Randomized Clinical Trials
Source: Nutrients. 2024 May 7;16(10):1402. doi: 10.3390/nu16101402 (PMC11124475; doi:10.3390/nu16101402)
Supplement: Supplementary file 1 [file nutrients-16-01402-s001.zip › nutrients-2964580-supplementary.pdf]

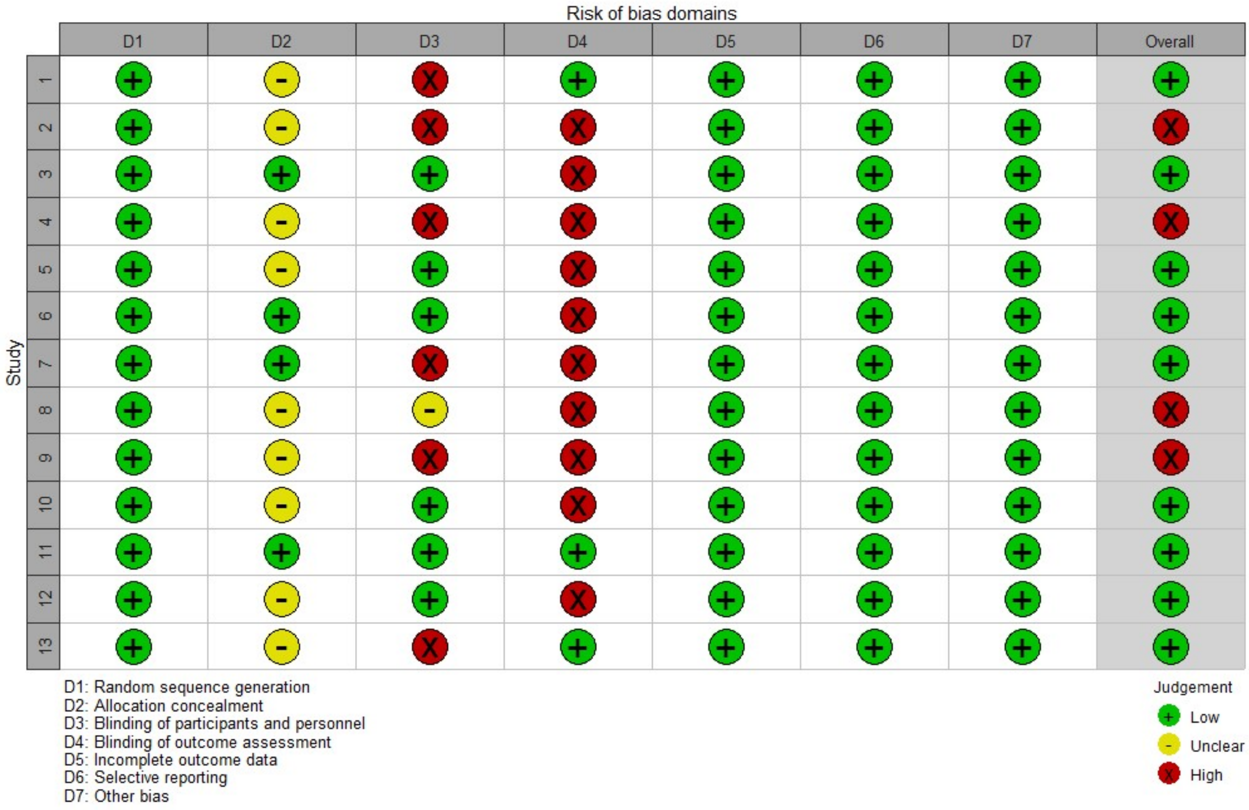

Figure S1. Risk of bias of included studies.

(1) Cannata-Andía et al., 2022; (2) Cervero et al., 2022; (3) Murai et al., 2021; (4) Karonova et al., 2022; (5) Jaun e al., 2023; (6) Bychinin et al., 2022; (7) Zurita-Cruz et al., 2022; (8) Sarhan et al., 2022; (9) Annweiler et al., 2022; (10) Mariani et al., 2022; (11) De Niet et al., 2022; (12) Maghbooli et al., 2021; (13) Domazet Bugarin et al.,2023.

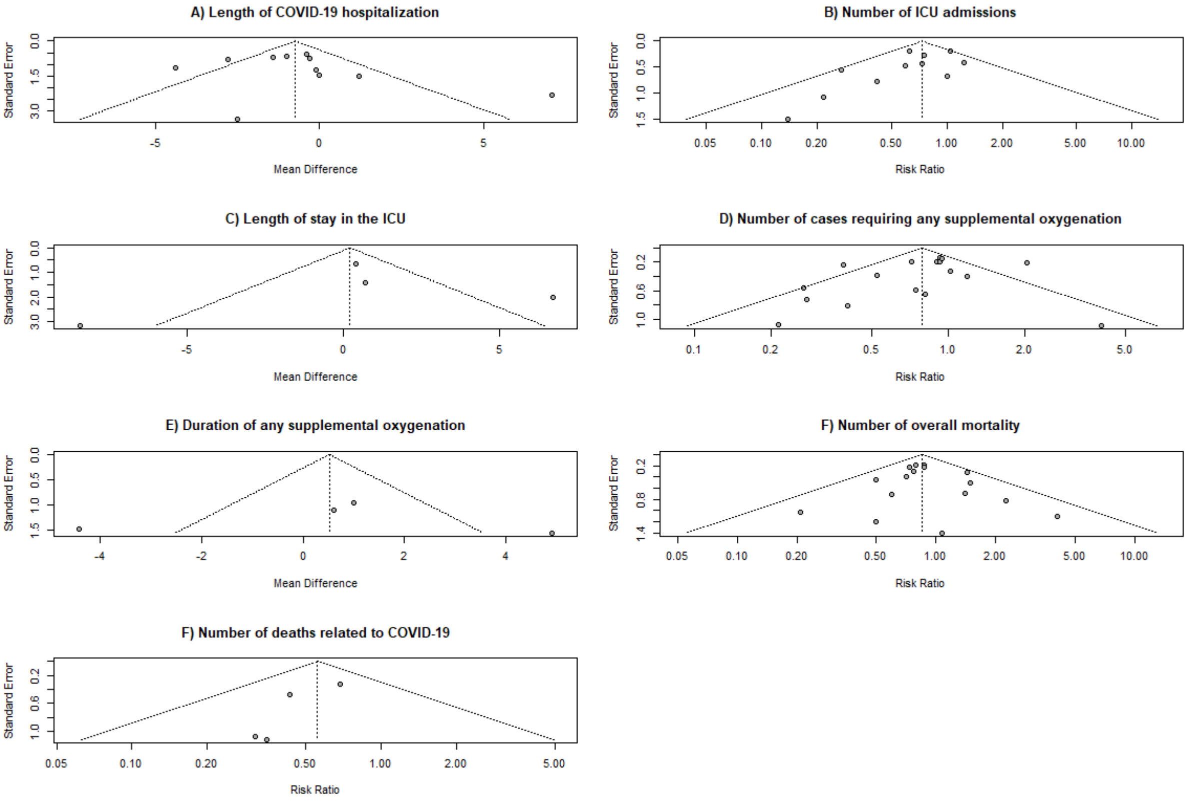

Figure S2. Funnel plots for the associations of between vitamin D3 supplementation and COVID-19.
